# On the improvement of CBCT image quality for soft tissue‐based SRS localization

**DOI:** 10.1002/acm2.12470

**Published:** 2018-10-07

**Authors:** Weihua Mao, Stephen J. Gardner, Karen Chin Snyder, Ning Winston Wen, Hualiang Zhong, Haisen Li, Paul Jackson, Mira Shah, Indrin J. Chetty

**Affiliations:** ^1^ Henry Ford Health System Detroit MI USA

**Keywords:** CBCT, imaging dose, noise, SRS

## Abstract

**Purpose:**

We explore the optimal cone‐beam CT (CBCT) acquisition parameters to improve CBCT image quality to enhance intracranial stereotactic radiosurgery (SRS) localization and also assess the imaging dose levels associated with each imaging protocol.

**Methods:**

Twenty‐six CBCT acquisition protocols were generated on an Edge^®^ linear accelerator (Varian Medical Systems, Palo Alto, CA) with different x‐ray tube current and potential settings, gantry rotation trajectories, and gantry rotation speeds. To assess image quality, images of the Catphan 504 phantom were analyzed to evaluate the following image quality metrics: uniformity, HU constancy, spatial resolution, low contrast detection, noise level, and contrast‐to‐noise ratio (CNR). To evaluate the imaging dose for each protocol, the cone‐beam dose index (CBDI) was measured. To validate the phantom results, further analysis was performed with an anthropomorphic head phantom as well as image data acquired for a clinical SRS patient.

**Results:**

The Catphan data indicates that adjusting acquisition parameters had direct effects on the image noise level, low contrast detection, and CNR, but had minimal effects on uniformity, HU constancy, and spatial resolution. The noise level was reduced from 34.5 ± 0.3 to 18.5 ± 0.2 HU with a four‐fold reduction in gantry speed, and to 18.7 ± 0.2 HU with a four‐fold increase in tube current. Overall, the noise level was found to be proportional to inverse square root of imaging dose, and imaging dose was proportional to the product of total tube current‐time product and the cube of the x‐ray potential. Analysis of the anthropomorphic head phantom data and clinical SRS imaging data also indicates that noise is reduced with imaging dose increase.

**Conclusions:**

Our results indicate that optimization of the imaging protocol, and thereby an increase in the imaging dose, is warranted for improved soft‐tissue visualization for intracranial SRS.

## 1 INTRODUCTION

The use of on‐board cone‐beam CT (CBCT) has led to significant improvement in localization accuracy for image‐guided radiation therapy. However, CBCT image quality generally falls short of helical CT in terms of low contrast visibility.^1^ This limits the application of CBCT in many instances to patient setup based on high contrast structures. Although skull matching is sufficient for the majority of intracranial stereotactic radiosurgery (SRS) treatment positioning, for a subset of cases (e.g., when the target abuts a sensitive structure or when deformation between simulation and treatment is more likely), improved soft‐tissue contrast is desired for enhancements in intracranial SRS localization. Image quality and imaging dose have been previously studied comparing different machines or existing acquisition CBCT protocols.^2^, ^3^, ^4^, ^5^, ^6^, ^7^, ^8^, ^9^, ^10^, ^11^, ^12^, ^13^, ^14^, ^15^, ^16^, ^17^, ^18^, ^19^ Elstrom et al. studied the imaging dose and quality for 100 and 125 kVp CBCT modes on a Varian Trilogy^®^ (Varian Medical Systems, Palo Alto, CA) linear accelerator.^8^ They found that contrast‐to‐noise ratio (CNR) increases with the square root of dose and their imaging dose measurement results indicate that imaging dose is proportional to the square of the tube potential with a same tube current‐time product. Default manufacturer preset acquisition protocols are typically designed to minimize imaging dose, and many institutions use the default protocols directly for treatment localization, potentially yielding suboptimal soft tissue visualization. Ding and Munro found that imaging doses due to Truebeam^®^ (Varian Medical Systems, Palo Alto, CA) CBCT scans are much less than conventional pair of orthogonal MV portal images. They reported that dose to the brainstem is 3.7, 0.24, and 0.16 cGy for a pair of MV portal imaging, Trilogy standard head CBCT, and Truebeam^®^ standard head CBCT scans respectively.^6^ This indicates that the Truebeam standard CBCT protocols deliver minimal imaging dose enabling potential to increase dose to improve soft‐tissue contrast. The Truebeam^®^ imaging platform makes it possible for users to adjust multiple acquisition parameters, including x‐ray tube potential, tube current‐time product, gantry rotation range, and gantry rotation speed, all of which may affect image quality.^20^, ^21^ This study explores sensitivity of image quality to all acquisition parameters and provides suggestions to enhance low contrast visibilities, specifically as it relates to intracranial SRS localization.

## 2 MATERIALS/METHODS

Twenty‐six CBCT acquisition protocols were generated for use on an Edge^®^ linac (Varian Medical Systems, Palo Alto, CA, USA) as listed in Table 1. All scans were designed utilizing the full‐fan bowtie filter. Imaging protocols with x‐ray potential settings of 80, 100, 125, and 140 kVp were used. The x‐ray tube current was varied between 15 and 126 mA. All protocols used the same pulse width of 20 ms and tube current‐time product was limited to 600 mAs or less except for the 80 kVp series. Gantry rotation speed was varied between 1.5°/s and 6.0°/s for half‐rotation trajectory scans (200° total scan angle), corresponding to a total projection number between 2000 and 500, and between 3.0°/s and 6.0°/s for full‐rotation trajectory scans (360° total scan angle), corresponding to total projection number between 1800 and 900.

**Table 1:** List of CBCT acquisition protocols and parameters evaluated in this study

Protocol name	Tube potential (kVp)	Tube current (mA)	Tube current‐time product (mAs)	Gantry rotation trajectory	Gantry speed (Deg/s)	Number of projections
Image Gently	80	10	100	Half	6.0	500
80kV_Half_15mA	80	15	150	Half	6.0	500
80kV_Half_60mA	80	60	600	Half	6.0	500
80kV_Half_126mA	80	126	1260	Half	6.0	500
80kV_Full_15mA	80	15	270	Full	6.0	900
80kV_Full_30mA	80	30	540	Full	6.0	900
80kV_Full_45mA	80	45	810	Full	6.0	900
80kV_Full_60mA	80	60	1080	Full	6.0	900
80kV_Full_70mA	80	70	1260	Full	6.0	900
Head	100	15	150	Half	6.0	500
Half_15mA_Slow	100	15	300	Half	3.0	1000
Half_15mA_VerySlow	100	15	450	Half	2.0	1500
Half_15mA_Slowest	100	15	600	Half	1.5	2000
Half_30mA	100	30	300	Half	6.0	500
Half_30mA_Slow	100	30	600	Half	3.0	1000
Half_45mA	100	45	450	Half	6.0	500
Half_60mA	100	60	600	Half	6.0	500
Full_15mA	100	15	270	Full	6.0	900
Full_15mA_Slow	100	15	540	Full	3.0	1800
Full_30mA	100	30	540	Full	6.0	900
125kV_Half_15mA	125	15	150	Half	6.0	500
125kV_Full_15mA	125	15	270	Full	6.0	900
125kV_Full_30mA	125	30	540	Full	6.0	900
140kV_Half_15mA	140	15	150	Half	6.0	500
140kV_Full_15mA	140	15	270	Full	6.0	900
140kV_Full_30mA	140	30	540	Full	6.0	900

### 2.A Imaging dose measurement

To evaluate imaging dose, the cone‐beam dose index (CBDI) was measured for all CBCT protocols using a 10 cm pencil chamber in a standard CT dose index (CTDI) head phantom (16 cm in diameter) (Computerized Imaging Reference System, Inc., Norfolk, VA, USA).^4^, ^9^ Doses at the central and four peripheral positions at 9:00, 12:00, 3:00, and 6:00 o'clock were measured for all half‐rotation acquisitions with specified rotation gantry between 20° to 180°E. Peripheral dose was calculated as

(1) $$D_{\mathrm{periphery}}=\left(D_{12}+D_{3}+D_{9}+D_{6}\right)/4$$

where *D*_12_, *D*_3_, *D*_9_, and *D*_6_ are the dose values measured at 12:00, 3:00, 9:00, and 6:00 o'clock position respectively. The weighted CBDI (wCBDI) for half rotation protocols were calculated as

(2) $$wCBDI_{\mathrm{half}}=\left(D_{\mathrm{center}}+2*D_{\mathrm{periphery}}\right)/3$$

while *D*_*center*_ is the dose at the phantom center. Due to rotational symmetry, the weighted CBDI for full‐rotation protocols were:

(3) $$wCBDI_{\mathrm{full}}=\left(D_{\mathrm{center}}+2*D_{12}\right)/3.$$

In order to compare wCBDI for CBCT protocols with different tube current‐time products, normalized cone‐beam dose index (nCBDI) was defined as the wCBDI per 100 mAs.

### 2.B Catphan phantom study

To evaluate image quality, a Catphan^®^ 504 phantom (The Phantom Laboratory, Salem, NY) was scanned using each CBCT protocol multiple times (3–6 image acquisitions for each protocol). The Catphan^®^ 504 phantom has four test modules: CTP404 for geometry and sensitometry, CTP528 for high resolution, CTP515 for low contrast, and CTP486 for uniformity as described in the website and manual.^22^ The CBCT images were reconstructed on the Edge^®^ treatment console using the following settings: Standard post‐processing smoothing filter, Medium ring correction factor, 1 mm slice thickness, 512 × 512 matrix resolution, and 0.51 mm pixel size. All reconstructed Catphan images were analyzed using a commercially available software package, Catphan QA program (Image Owl, Inc., Greenwich, NY). This software quantitatively evaluates the following imaging metrics:

1. Noise — defined as the standard deviation of measured HU values in the central region in the CTP 486 module. This module is a solid uniform cylinder with a designed CT number within 20 HU at standard scanning protocols. The central region has a diameter 40% of the module diameter.
2. Low contrast detectability — based on the smallest detected diameter of inserts at 1% contrast; inserts have different diameters (2 to 9 mm and 15 mm) in the CTP 515 Module. For each target size, two rows of circle ROIs were generated in the background inside and outside of the inserts. The average HU was calculated for every circle and the standard deviation (SD) was calculated from the set of average HU numbers. The insert of a ce f its standard deviation of average HU numbers is less than 1% contrast (=10 HU)

(4) 4×SD≤10HU

- Uniformity — based on average HU value of each peripheral cylinder ($I_{n},n=1\;to\;4$) compared with that of a central cylinder (*I*_*ctr*_) in the CTP 486 Module.

(5) $$Uniformity=\max\{|I_{n}-I_{\mathrm{ctr}}|\},n=1\;\text{to}\;4.$$

- HU Constancy — maximum absolute difference between measured CT numbers ($I_{n},n=1\;to\;3$) from expected CT numbers ($I_{n}^{\mathrm{expect}},n=1\;to\;3$) for three known inserts in the CTP 404 Module. These inserts are air (−1000 HU), LDPE (−100 HU) and acrylic (120 HU).

(6) $$HU\;Constancy=\max\{|I_{n}-I_{n}^{\mathrm{expect}}|\},n=1\;\text{to}\;3.$$

- Spatial resolution — based on the Modulation transfer function (MTF) of two embedded BBs, with average frequencies listed at 50% and 10% MTF levels separately in the CTP 528 Module of the Catphan 504 phantom.
- Contrast‐to‐noise ratio (CNR) — based on a cylinder with a diameter of 15 mm and a length of 40 mm, at 1% contrast level. A region of interest (ROI) cylinder was contoured at the center of the 15 mm 1% contrast insert. A background (BKG) cylinder was contoured adjacent to the insert. Both contoured volumes were propagated to all image sets after image registrations within Eclipse^TM^ (Varian Medical Systems, Palo Alto, CA). Average HU values (*I*_*ROI*_ and *I*_*BKG*_) and standard deviations (*N*_*ROI*_ and *N*_*BKG*_) were calculated within the ROI and background contours respectively. CNR was calculated by the following equation^20^, ^21^:
  (7) $$CNR=\frac{I_{\mathrm{ROI}}-I_{\mathrm{BKG}}}{N_{\mathrm{BKG}}}$$

### 2.C STEEV phantom study

**Figure 1:**
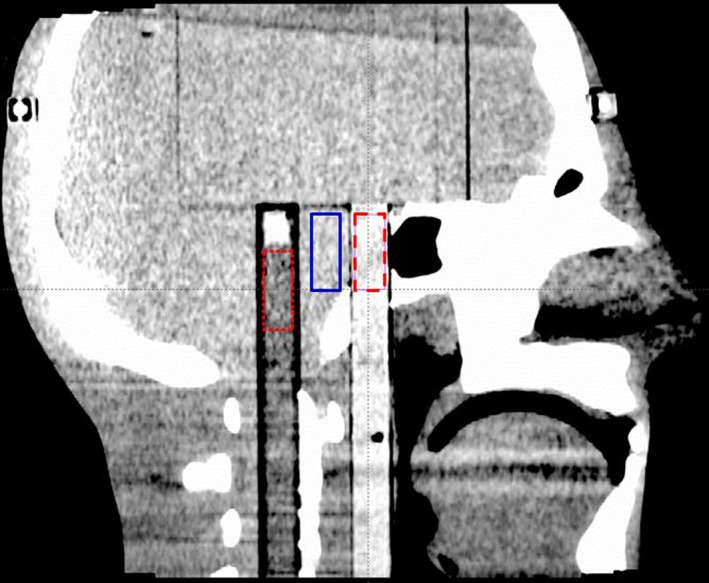
Sagittal view of the STEEV phantom. Posterior volume contoured in the water tube demonstrated by red dotted line, the center volume contoured in the background as a reference demonstrated by solid blue line, and the anterior volume contoured in the acrylic rod demonstrated by the pink dashed line.

An anthropomorphic head phantom (STEEV, Computerized Imaging Reference Systems, Inc., Norfolk, VA) was also used to evaluate CNR. This phantom is constructed of tissue‐equivalent materials to simulate soft tissues and bones. A water tube and an acrylic rod were inserted into the phantom as shown in Fig. 1. Three cylinders (10 mm diameter and 25 mm length) were contoured in the water tube, acrylic rod, and background. The noise level and CNR were calculated from the ROI in the water and acrylic inserts for each set of images.

### 2.D Patient data study

The 100 kVp high quality full‐rotation CBCT (Full_30mA and wCBDI = 1.18 cGy) was used for initial position of an intracranial radiosurgery patient while both a 100 kVp full‐rotation (Full_15mA) and a half‐rotation (Head) was used to verify the patient after shifts were applied. One region of interest with a volume of 0.15 cm^3^ was contoured in the ventricle and a similar region was contoured as background in the nearby brain parenchyma. Both contours were propagated to the simulation CT and each CBCT image set based on image registration, and noise level and CNR were calculated.

## 3 RESULTS

### 3.A Imaging dose results

**Figure 2:**
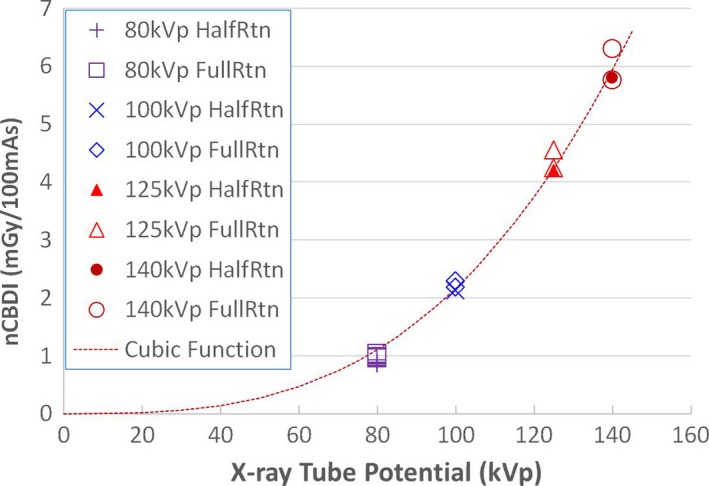
Normalized weighted cone‐beam dose index (nCBDI) as a function of x‐ray tube potential. Curves were fitted by cube of tube potential.

The wCBDI measurement results are listed in Table 2. As expected, the imaging dose increases with tube potential, as illustrated in Fig. 2. The normalized weighted cone‐beam dose index (nCBDI) as a function of tube potential were best fitted with cube of the tube potential. The normalized cross correlation coefficient between normalized weighted cone‐beam dose index and cube of maximum tube potential was 0.992. Relative to the curve fit, the maximum deviation was 0.16 ± 0.06 mGy/100 mAs or about 20% for 80 kVp protocols while the deviations for other potential settings were less than 7%.

**Table 2:** Summary of the Catphan phantom study image quality results. Standard deviations are listed in parentheses

Protocol name	wCBDI (cGy)	Noise (HU)	Low contrast (mm)	CNR	MTF 10% (lp/cm)	MTF 50% (lp/cm)	Uniformity (HU)	HU constancy (HU)
Image gently	0.09	62.4 (0.8)	>=15	0.20 (0.3)	4.4 (0.5)	7.0 (0.3)	8.3 (3.3)	18.8 (9.1)
80kV_Half_15mA	0.14	50.2 (0.3)	>=15	0.27 (0.2)	3.9 (0.2)	6.7 (0.1)	7.5 (0.5)	8.3 (4.0)
80kV_Half_60mA	0.52	25.1 (0.3)	9 ~ 15	0.58 (0.5)	4.2 (0.2)	7.2 (0.2)	5.7 (2.3)	11.0 (3.5)
80kV_Half_126mA	1.09	17.9 (0.3)	6	0.91 (0.1)	4.1 (0.1)	7.0 (0.2)	5.1 (1.1)	9.3 (0.6)
80kV_Full_15mA	0.28	36.5 (0.2)	8 ~ 9	0.39 (0.3)	4.2 (0.1)	7.2 (0.1)	3.7 (2.0)	9.0 (4.6)
80kV_Full_30mA	0.53	25.0 (0.2)	7	0.49 (0.1)	4.1 (0.2)	7.0 (0.2)	2.0 (0.2)	22.7 (1.2)
80kV_Full_45mA	0.79	20.7 (0.3)	6	0.66 (0.2)	4.1 (0.1)	7.1 (0.1)	3.3 (1.4)	13.7 (2.5)
80kV_Full_60mA	1.04	18.0 (0.2)	5 ~ 6	0.74 (0.2)	4.1 (0.1)	7.0 (0.1)	2.5 (0.9)	14.0 (3.0)
80kV_Full_70mA	1.22	16.6 (0.0)	5	0.80 (0.2)	4.1 (0.1)	7.1 (0.1)	3.7 (0.9)	14.0 (1.0)
Head	0.32	34.5 (0.3)	>=15	0.43 (0.4)	4.0 (0.2)	7.0 (0.1)	7.5 (1.3)	10.6 (3.7)
Half_15mA_Slow	0.63	24.9 (0.3)	>=15	0.56 (0.3)	4.1 (0.1)	7.0 (0.1)	7.4 (2.2)	11.9 (1.0)
Half_15mA_VerySlow	0.95	20.7 (0.1)	>= 15	0.91 (0.3)	4.1 (0.1)	7.1 (0.1)	6.2 (0.5)	12.0 (3.7)
Half_15mA_Slowest	1.27	18.5 (0.2)	>= 9	0.79 (0.3)	4.1 (0.0)	7.0 (0.0)	6.3 (1.8)	12.5 (1.8)
Half_30mA	0.63	25.5 (0.2)	9	0.66 (0.2)	4.0 (0.3)	6.9 (0.3)	6.3 (3.0)	13.0 (3.6)
Half_30mA_Slow	1.27	18.3 (0.2)	7 ~ 8	0.90 (0.4)	4.1 (0.1)	7.1 (0.1)	3.9 (0.9)	9.8 (3.1)
Half_45mA	0.95	21.0 (0.2)	8	0.78 (0.3)	4.0 (0.1)	7.0 (0.1)	3.8 (0.5)	13.0 (1.4)
Half_60mA	1.27	18.7 (0.2)	7	0.93 (0.1)	4.1 (0.0)	7.1 (0.1)	3.4 (1.5)	13.0 (4.1)
Full_15mA	0.62	24.6 (0.3)	7	0.58 (0.2)	4.2 (0.2)	7.1 (0.2)	2.5 (1.5)	5.0 (2.6)
Full_15mA_Slow	1.09	17.6 (0.1)	5	0.87 (0.1)	4.0 (0.1)	7.0 (0.1)	2.8 (0.4)	2.5 (0.0)
Full_30mA	1.18	17.9 (0.1)	5	0.86 (0.2)	4.2 (0.0)	7.2 (0.0)	1.6 (0.8)	6.0 (2.6)
125kV_Half_15mA	0.63	26.5 (0.3)	9 ~ 15	0.55 (0.1)	4.1 (0.0)	7.1 (0.0)	5.9 (2.0)	20.5 (2.6)
125kV_Full_15mA	1.23	18.7 (0.3)	5	0.75 (0.2)	4.1 (0.1)	7.0 (0.1)	4.1 (0.1)	8.8 (2.8)
125kV_Full_30mA	2.29	13.6 (0.2)	4 ~ 5	1.09 (0.3)	4.1 (0.1)	7.0 (0.1)	3.9 (1.2)	10.7 (3.1)
140kV_Half_15mA	0.87	23.9 (0.2)	9 ~ 15	0.53 (0.1)	4.0 (0.1)	7.0 (0.1)	8.5 (2.8)	17.0 (4.4)
140kV_Full_15mA	1.70	16.7 (0.2)	5	0.69 (0.3)	4.2 (0.0)	7.1 (0.0)	4.9 (0.7)	8.8 (2.8)
140kV_Full_30mA	3.11	12.3 (0.1)	5	0.90 (0.2)	4.1 (0.0)	7.1 (0.1)	5.7 (1.5)	9.4 (0.9)

**Table 3:** Patient data image qualities of CBCT and simulation CT protocols

Protocol	Tube current‐time product (mAs)	Imaging dose (cGy)	ROI (HU)		Background (HU)		CNR
			Average	Noise	Average	Noise	
Sim CT	142	5.94	6.5	6.7	28.3	7.5	3.24
Head	150	0.32	−61.8	37.3	−26.4	35.4	0.95
Full_15mA	270	0.62	−28.5	28.1	4.2	27.6	1.16
Full_30mA	540	1.18	6.2	18.1	25.7	19.2	1.08

### 3.B Catphan phantom study results

Image quality results of the Catphan are summarized in Table 2. There is no clear correlation between the scan acquisition protocol settings and geometric distortion, spatial resolution, uniformity, or HU constancy. Noise decreases with number of projections with fixed x‐ray current setting for 100 kVp protocols. Relative to the default half‐rotation scan (15 mA at 6°/s), noise is decreased from 34.5 ± 0.3 HU to 18.5 ± 0.2 HU with ultra‐slow rotation (1.5°/s), which utilizes four times as many projections. Noise of the default full‐rotation scan (15 mA at 6°/s) is decreased from 24.6 ± 0.3 HU to 17.6 ± 0.1 HU with slow rotation (3°/s) with twice as many projections acquired.

**Figure 3:**
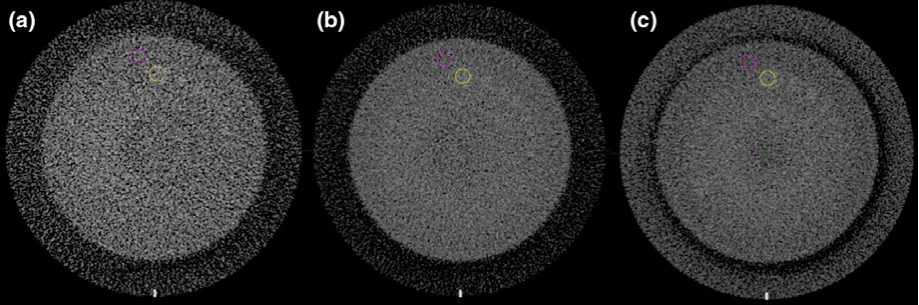
Comparison of the Catphan low contrast module scanned with different CBCT protocols. (a) standard half‐rotation 100 kVp 15 mA scan (wCBDI — 0.3 cGy); (b) full‐rotation 100 kVp 30 mA scan (wCBDI — 1.2 cGy); (c) full‐rotation 80 kVp 70 mA scan (wCBDI — 1.2 cGy). HU window: [0, 300 HU]. Two contours are for contrast‐to‐noise ratio measurements.

For low contrast object detection, improvement in visualization (better detection of small low contrast objects) is aided by a decrease image noise. Figure 3 shows a side‐by‐side comparison of CTP515 Low Contrast Module images for 3 CBCT protocols evaluated. When the tube current‐time product was increased by a factor of 4, low contrast detection was enhanced from 9 mm to either 6 mm or 5 mm as shown in Fig. 3. The CNR for 1% contrast level increased from 0.43 ± 0.04 to 0.93 ± 0.01 when the tube current‐time product was increased by four times by increasing x‐ray tube current or to 0.93 ± 0.03 by slowing down gantry rotation.

**Figure 4:**
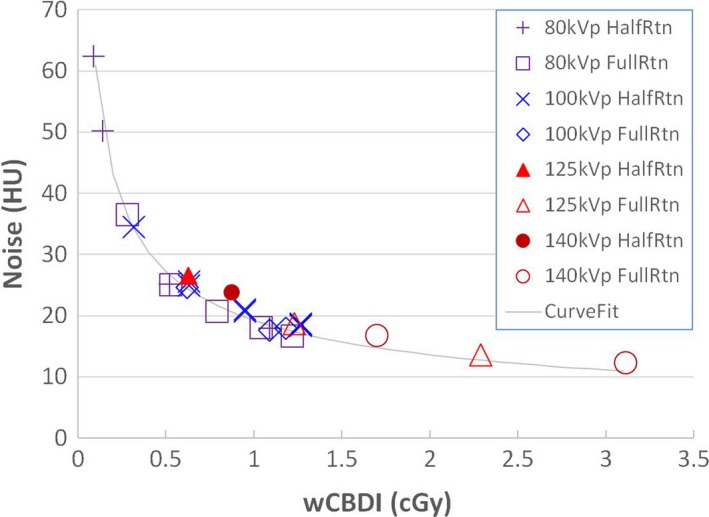
Noise as a function of weighted cone‐beam dose index (wCBDI). Noise was fitted as an inverse square root function of wCBDI.

**Figure 5:**
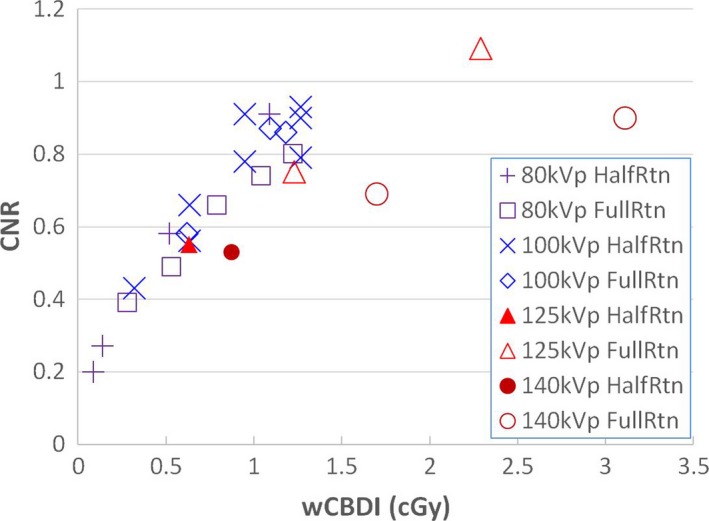
Catphan contrast‐to‐noise ratio as a function of weighted cone‐beam dose index (wCBDI).

As shown in Fig. 4, image noise was directly correlated with imaging dose (wCBDI) with a standard deviation of 1.4 HU. Consequently, a higher tube potential setting resulted in an increase in imaging dose with a corresponding decrease in image noise. The relationship between noise and wCBDI was best fitted by an inverse square root function (Noise ~ $1/\sqrt{\mathrm{wCBDI}}$ ). The normalized cross correlation coefficient between noise and $1/\sqrt{w\text{CBDI}}$ was 0.990. The contrast‐to‐noise ratio is plotted as a function of the wCBDI in Fig. 5. The CNR increases with imaging dose, and protocols of 80 and 100 kV were found to yield the largest CNR.

### 3.C STEEV phantom study results

**Figure 6:**
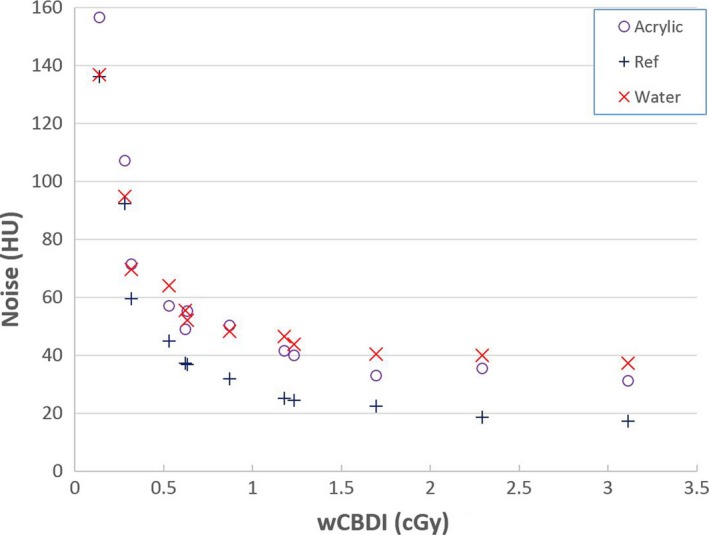
Noise results of the STEEV phantom CBCT image for three volumes as functions of imaging dose (wCBDI).

**Figure 7:**
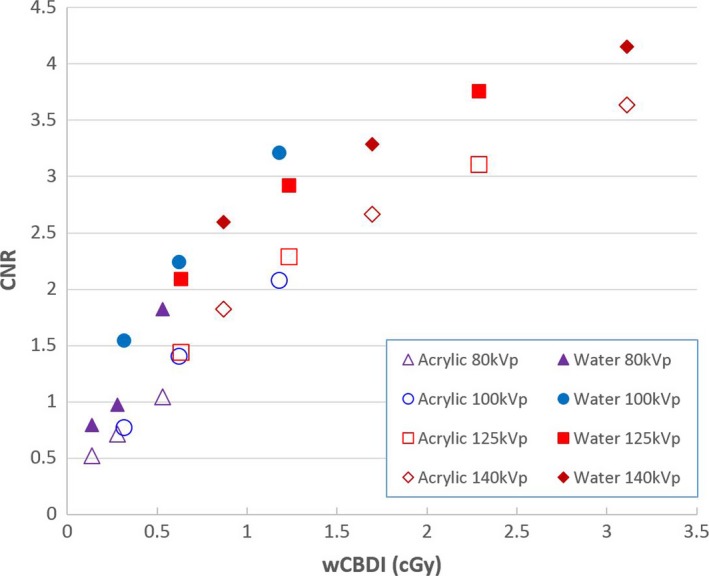
Contrast‐to‐Noise Ratio results of the STEEV phantom images with different tube potentials as functions of imaging dose (wCBDI).

Noise values for three different regions of interest in STEEV phantom images scanned utilizing the 12 different protocols are illustrated in Fig. 6 as functions of the imaging dose. Contrast‐Noise‐Ratio was calculated for Acrylic and Water volumes compared with the reference volume for every scan. CNR results for both Acrylic and Water are shown in Fig. 7 for different x‐ray tube potential settings.

### 3.D Patient data results

**Figure 8:**
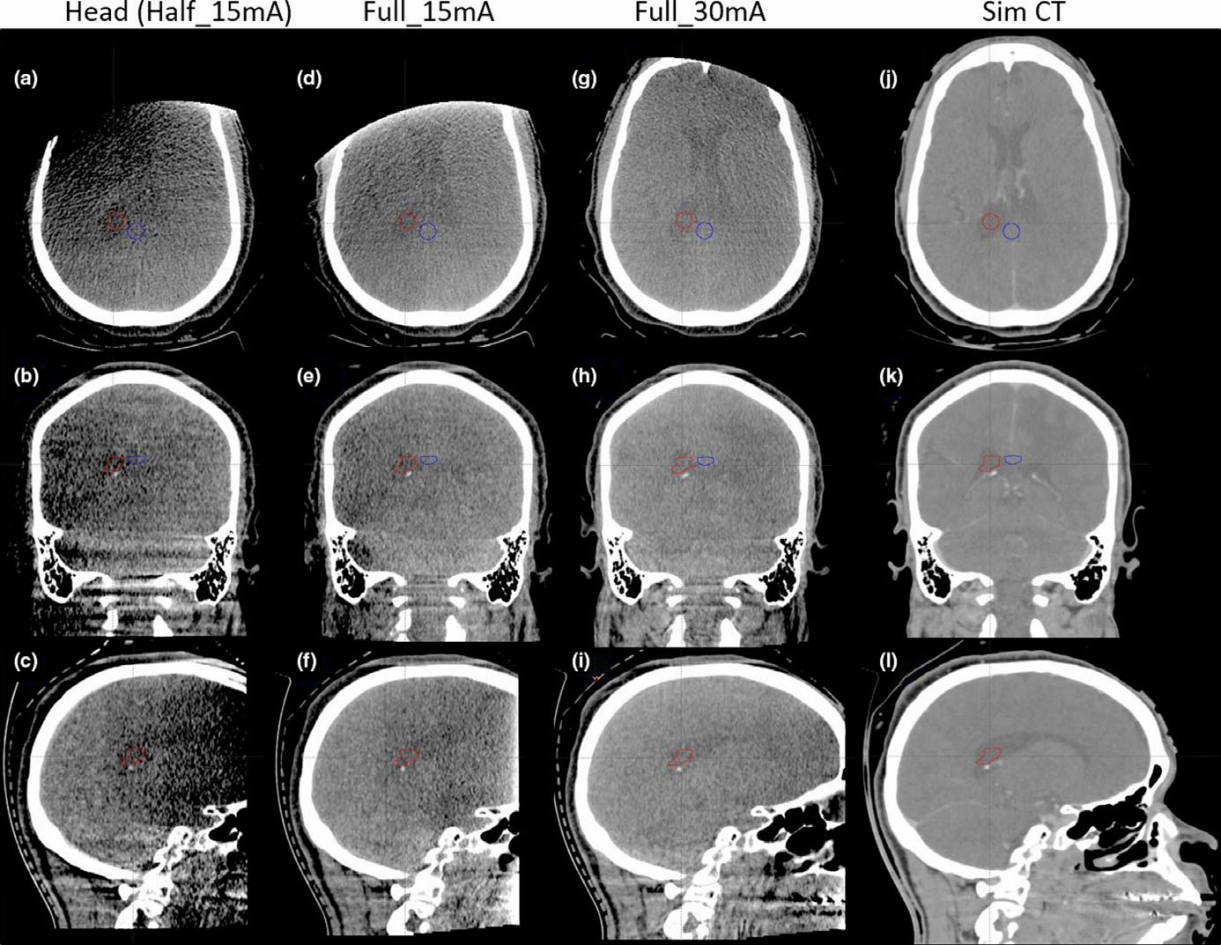
Images of standard 100 kV half‐rotation (Head, wCBDI = 0.32 cGy) (a, b, c), full‐rotation (Full_15mA, wCBDI = 0.62 cGy) (d, e, f), high quality (Full_30mA, wCBDI = 1.18 cGy) (g, h, i), and simulation CT (j, k, l) for a SRS patient. HU window: [−100, 300 HU]. Region of interest and back ground were contoured for CNR calculation.

Figure 8 displays images of an intracranial SRS patient scanned with the following three settings: 100 kV high quality CBCT (Full_30mA and wCBDI = 1.18 cGy); standard full‐rotation CBCT (Full_15mA and wCBDI = 0.62 cGy); and half‐rotation CBCT (Head and wCBDI = 0.32 cGy). Simulation CT images are also shown for comparison, and all images are displayed at the same window/level settings. Relative to the standard half‐rotation image, noise of high quality CBCT decreased from 37 to 18 HU and from 35 to 19 HU for volumes contoured in the ventricle and background respectively (as listed in Table 3).

## 4 DISCUSSION

The traditional CTDI is measured by a 10 cm length ion chamber in a narrow beam of a width of nominal slice thickness and results are normalized by the width. The CBDI was measured by the 10 cm length ion chamber in an open field as in CBCT acquisitions. We have compared the CTDI and CBDI for eight CBCT modes (both half rotation and full rotation for four different voltage settings from 80 to 140 keV). The average ratio between CBDI and CTDI results is 1.02 with a standard deviation of 0.08. It is understandable since the open field beams of CBDI measurements will result in slightly more scatter than narrow beams of CTDI measurement.

It is well established that CBCT imaging noise is reduced by increasing the tube current‐time product. Here we have quantified that image noise is reduced and low contrast detection is increased by either of the following three approaches: (a) slowing down the gantry rotation or expanding the gantry rotation range to acquire more projections, (b) increasing the tube current‐time product, or (c) increasing the tube potential. Based on our knowledge, there has been no comprehensive comparison of imaging doses at different tube potential settings in the CBCT setting. Elstrom et al. reported on a Varian Trilogy linear accelerator that weighted CTDI was 31.8 and 86.7 mGy for a half rotation CBCT protocol (100 kV and 744 mAs) and a full rotation CBCT protocol (125 kV and 1338 mAs), respectively,^8^ They also found the ratio of normalized CTDI of 125 over 100 kV scans to be approximately 1.5, which was proportional to the square of the tube potential (1.56) with a constant tube current‐time product. Based on the work of Islam et al. for point dose measurement results of CBCT scans on an Elekta Synergy linear accelerator, the imaging dose ratio between 120 and 100 kV CBCT scans for the same tube current‐time product (660 mAs) can be computed from Table 2 of Ref. 10 Their value of 1.70 ± 0.03 is very similar to the cube of the potential ratio (1.73). Our dose measurement shows that the ratio of normalized weighted CBDI between 125 and 100 kV CBCT protocols is 1.96 ± 0.16 while the cube of potential ratio is 1.95.

Our results indicate that the imaging dose is the single largest determinant of image noise. Quantitatively, the CBCT imaging noise is proportional to the inverse square root of the imaging dose (wCBDI). Increasing the tube potential leads to less imaging noise; however, this will also result in increased imaging dose and less contrast between different tissue types at the same time, thereby potentially compromising the contrast‐to‐noise ratio. Therefore, our results support the use of lower tube potential settings (80 or 100 kVp) as the preferred technique for CBCT imaging of the brain. To maintain acquisition efficiency, increasing the x‐ray tube current‐time product is more promising as compared to increasing the number of projections acquired to increase soft tissue contrast. The selection of a CBCT imaging technique protocol is a balance between imaging dose and localization accuracy. Default manufacturer CBCT acquisition protocols were designed with minimal patient dose in mind. SRS patients will benefit from better quality CBCT imaging contrast afforded with slightly higher imaging dose. Clinically, this will improve visual detection of soft tissues necessary for accurate visualization and localization. Other improvements associated with better soft tissue contrast include contouring, dose calculation, and deformable image registration, which may facilitate online adaptive radiation therapy in SRS treatment.

## 5 CONCLUSIONS

Better soft‐tissue visualization in the context of intracranial SRS can be achieved through optimization of CBCT imaging protocols, with a moderate increase in the imaging dose relative to standard manufacturer settings.

## CONFLICT OF INTERESTS

None declared.
